# Loss of CDYL Results in Suppression of CTNNB1 and Decreased Endometrial Receptivity

**DOI:** 10.3389/fcell.2020.00105

**Published:** 2020-02-25

**Authors:** Xiaowei Zhou, Bufang Xu, Dan Zhang, Xiaoping Jiang, Hsun-Ming Chang, Peter C. K. Leung, Xiaoyu Xia, Aijun Zhang

**Affiliations:** ^1^Department of Reproductive Medical Center, Ruijin Hospital, School of Medicine, Shanghai Jiao Tong University, Shanghai, China; ^2^Department of Obstetrics and Gynecology, Chinese People’s Armed Police Force Shanghai Corps Hospital, Shanghai, China; ^3^Department of Obstetrics and Gynaecology, BC Children’s Hospital Research Institute, The University of British Columbia, Vancouver, BC, Canada; ^4^Department of Histoembryology, Genetics and Developmental Biology, School of Medicine, Shanghai Jiao Tong University, Shanghai, China; ^5^Shanghai Key Laboratory of Reproductive Medicine, Shanghai Jiao Tong University, Shanghai, China

**Keywords:** recurrent implantation failure, chromodomain Y like, catenin beta 1, migration, endometrial receptivity

## Abstract

Impaired endometrial receptivity is one of the major causes of recurrent implantation failure (RIF), although the underlying molecular mechanism has not been fully elucidated. In the present study, we demonstrated that chromodomain Y like (CDYL) was highly expressed in the endometrium at mid-secretory phase during the normal menstrual cycles. However, the expression of CDYL was downregulated in the endometrial tissues obtained from women with RIF, consistently with the protein level of LIF, which is a marker of endometrial receptivity. In *CDYL*-knockdown human endometrial Ishikawa cells, we identified 1738 differentially expressed genes (DEGs). Importantly, the *catenin beta 1* (*CTNNB1*) expression was dramatically reduced responding to the *CDYL* inhibition, both in Ishikawa cells as well as the primary endometrial epithelial and stromal cells. In addition, the expression of *CTNNB1*was decreased in the endometrium from RIF patients as well. These results suggested that the expression of CTNNB1 was regulated by CDYL in endometrium. The cell migration was impaired by *CDYL*-knockdown in Ishikawa cells and primary endometrial stromal cells (ESCs), which could be rescued by *CDYL* or *CTNNB1* overexpression. Collectively, our findings indicated that the decreased expression of CDYL may suppress endometrial cell migration capability by affecting CTNNB1 expression, which would contribute to poor endometrial receptivity in women with RIF.

## Introduction

Embryo implantation is a profound process composing of embryo apposition, adhesion, penetration, and invasion (Singh et al., 2011). Recurrent implantation failure (RIF) is defined as the absence of implantation after either transfer of three or more morphologically good quality embryos or placement of 10 or more embryos in several transfers (Koot et al., 2019). RIF has become an obstacle in assisted reproductive techniques and accounts for approximately 10% of women who fail to achieve a clinical pregnancy during their *in vitro* fertilization-embryo transfer (IVF-ET) treatment (Polanski et al., 2014; Macklon, 2017; Nowak et al., 2017). The etiologies for RIF are complex that involve multiple factors, including disturbed immune system, intrauterine adhesion, and poor endometrial receptivity. Among these factors, poor endometrial receptivity is the main cause for RIF (Timeva et al., 2014; Vlachadis et al., 2014; Craciunas et al., 2019). There have been researches using either microarray technology or to identify the transcriptomic signature of endometrial samples from RIF patients (Mahajan, 2015; Zhou et al., 2019). However, the pathogenesis of poor endometrial receptivity in RIF cases has not been fully elucidated yet.

Endometrial receptivity generally refers to the competence of the endometrium to accept an embryo during the implantation window (Miravet-Valenciano et al., 2015). Once the embryo forms the firm adhesion sites in the endometrial epithelium, the trophoblasts penetrate the epithelium and subsequently deeply infiltrate the maternal decidua stroma. Trophoblast cells proliferate, differentiate, and connect with the maternal vasculature to form a functional placenta (Robins, 2016; Zhang et al., 2018). During this process, the migration capability of endometrial epithelial cells (EECs) is enhanced, that EECs undergo apoptosis and migrate away from the implantation site, make a space for trophoblast penetration (Garrido-Gomez et al., 2012; Cui et al., 2018). At the same time, the migration of endometrial stromal cells (ESCs) is significantly increased, in order to accommodate the invading trophoblast. Inhibiting the motility of ESCs suppressed trophoblast invasion into the ESC monolayer (Grewal et al., 2008, 2010). Taken together, in endometrial cells, the dynamic regulation of migration ability is important for building endometrial receptivity. However, the molecular mechanism involved is largely unclear.

Human *Chromodomain Y* (*CDY*) genes are expressed specifically in the testis and implicated in male infertility. Although the Y-linked *CDY* genes are testis-specific, the autosomal *Chromodomain Y like* (*CDYL*) gene is ubiquitously expressed in many tissues (Lahn and Page, 1999; Ghorbel et al., 2014), probably regulated by sex hormones (Gill-Sharma et al., 2012). Functionally, CDYL has been reported involved in the regulation of cell migration. For instance, *CDYL* silencing has been shown to inhibit neuronal migration by transcriptionally repressing *RhoA* (Qi et al., 2014; Qin et al., 2017; Schoeler et al., 2018). In germline conditional knockout mice, targeted depletion of *Cdyl* led to a phenotype of teratozoospermia and infertility, indicating a crucial role of *Cdyl* in regulating spermatogenesis and male fertility (Xia et al., 2019). However, there has not been scientific report about the functions of *CDYL* in female fertility. In our previous studies, using the genome-wide expression profiling analysis, we verified that the expression of PECAM1 and TGF-β1 was clearly reduced in the mid-secretory endometrium of RIF group, which may contribute to the poor endometrial receptivity (Guo et al., 2018). Similarly, we found that expression of *CDYL* was significantly downregulated in the endometrium from women with RIF. These findings proposed that the decreased expression of *CDYL* may contribute to the poor endometrial receptivity, which would cause the occurrence of RIF. In this study, we explored the impact of inhibited expression of *CDYL* on endometrial cell behaviors, as well as the underlying molecular mechanisms.

## Materials and Methods

### Ethical Approval

All participants signed informed consent forms, and studies of human subjects were approved by the Institutional Ethics Committee of Ruijin Hospital, Shanghai Jiao Tong University, School of Medicine.

### Participants and Endometrial Biopsies

Biopsies were collected at the Reproductive Medical Center of Ruijin Hospital from December 2015 to December 2019. A total of 44 mid-secretory phase endometrial specimens (Table 1) were obtained from the RIF patients (*n* = 22) and controls (*n* = 22) at day LH + 7 using pipe suction curettage (LILYCLEANER, Shanghai, China). Women with a history of no pregnancy after at least three times of embryo transfers (including a total of ≥four good-quality embryos) were identified as RIF patients. Women suffering from secondary infertility with tubal factor (tubal obstruction factor on hysterosalpingography or having tubal ligation) who underwent a clinical pregnancy after the first embryo transfer were assigned as controls. Tubal factor infertility cases caused by hydrosalpinx, salpingitis, etc., were excluded. The inclusion and exclusion criteria for both groups were the same as described previously (Guo et al., 2018; Xu et al., 2018). In addition, for the isolation of primary EECs and ESCs, another 12 mid-secretory phase endometrial samples from controls were obtained (Grewal et al., 2008).

**TABLE 1:** Demographic characteristics of the women in control and RIF groups recruited in this study.

**Variable**	**Control (*n* = 22)**	**RIF (*n* = 22)**	***P***
Age (years)	30.68 ± 3.83	31.63 ± 3.81	0.412
BMI (kg/m^2^)	22.05 ± 2.11	21.35 ± 1.72	0.251
Basal FSH level (mIU/mL)	6.48 ± 1.52	6.62 ± 1.99	0.804
Basal LH level (mIU/mL)	4.04 ± 2.26	3.22 ± 1.71	0.187
Basal E_2_ level (pg/mL)	37.21 ± 13.04	39.18 ± 18.84	0.693
Times of embryo transfers	1 (1,1)	4 (3,10)	<0.001
Number of embryos per transfer	1.68 ± 0.48	1.69 ± 0.47	0.972
Average score of day 3 embryos transferred	7.49 ± 0.69	7.69 ± 0.74	0.144
Score of transferred blastocysts	(0/0, 0%)	3BB (16/65, 25%) 4AB (26/65, 40%) 4BB (23/65, 35%)	

*Data presented as mean ± SD or frequency. The difference between control and RIF patients was analyzed by an independent samples *t*-test except “times of embryo transfers,” which was calculated by independent-samples Mann–Whitney *U*-test (median, range). BMI, body mass index; FSH, follicle stimulating hormone; LH, luteinizing hormone; E_2_, estradiol.*

At the same time, 20 endometrial tissues of proliferative phase were staged and obtained from controls as follows (Table 2): early proliferative (cycle day 3–4), *n* = 8; late proliferative (cycle day 11–13), *n* = 12.

**TABLE 2:** Demographic characteristics of the women in proliferative phase recruited in this study.

**Variable**	**Early proliferative phase (*n* = 8)**	**Late proliferative phase (*n* = 12)**	***P***
Age (y)	29.13 ± 3.72	29.25 ± 3.93	0.944
BMI (kg/m^2^)	22.40 ± 1.49	22.10 ± 3.05	0.798
Basal FSH level (mIU/mL)	6.95 ± 1.30	7.15 ± 2.36	0.831
Basal LH level (mIU/mL)	5.48 ± 2.71	4.62 ± 1.47	0.209
Basal E_2_ level (pg/ml)	43.38 ± 18.57	40.58 ± 12.21	0.688

*Data presented as mean ± SD. The differences of age, BMI, basal FSH, LH and basal E_2_ levels between three menstrual phase groups were analyzed by an independent samples *t*-test. BMI, body mass index; FSH, follicle-stimulating hormone; LH, luteinizing hormone; E_2_, estradiol.*

### RNA Extraction and RT-qPCR

Total RNA was extracted using the TaKaRa MiniBEST Universal RNA Extraction Kit according to the manufacturer’s instructions (Cat No. 9767, Takara, Tokyo, Japan). The quality of RNA was verified by the absorbance ratio of A260/230 and A260/280 using NanoDrop ND-1000 (Thermo Fisher, Waltham, MA, United States). For reverse transcription, 1.0 μg of RNA per sample was reverse transcribed using an Oligo (dT) primer and PrimeScript RT Enzyme Mix (Takara). The quantitative real-time PCR reaction was prepared using the SYBR Premix Ex Taq Kit (Takara) and performed using an ABI 7500 System (Life Tech, Applied Biosystems, Beverly, MA, United States). Each reaction included an initial denaturation step at 95°C for 30 s, followed by 40 cycles of amplification at 95°C for 5 s and annealing at 60°C for 34 s. The primers used in the experiments are listed in Supplementary Table S1. All assays were run in triplicates. Relative quantification of the mRNA levels was calculated based on the comparative cycle threshold (Ct) method with *GAPDH* as the endogenous control.

### Protein Preparation and Western Blot

Proteins were extracted by lyzing frozen endometrial samples using electric homogenizer GH-B (HYQ, United States), and cells with the radio immunoprecipitation assay (RIPA) lysis buffer (Thermo Fisher Scientific) supplemented by 1% protease inhibitor cocktail (Roche, Basel, Switzerland). The lysates were then centrifuged at 12,000 × *g* for 10 min at 4°C to collect the supernatant. Protein concentrations were measured using the BCA protein assay kit (Beyotime, China). Equal amounts of proteins (30 μg) were separated using 10% sodium dodecyl sulfate–polyacrylamide gel electrophoresis (SDS-PAGE) and transferred to polyvinylidene difluoride membranes (Millipore, Billerica, MA, United States). Membranes were blocked in 5% non-fat milk in Tris-buffered saline and 0.1% Tween 20, probed with protein-specific primary antibodies, incubated with HRP-conjugated secondary antibodies, and visualized via enhanced chemiluminescence (Millipore). Primary antibodies used in this study are listed in Supplementary Table S2. Quantification was performed using the built-in functions of Image J 1.46r (National Institutes of Health, United States) and normalized to GAPDH (Gallo-Oller et al., 2018).

### Immunohistochemistry and Immunofluorescence

Primary endometrial tissue samples were fixed with 4% paraformaldehyde (PFA); 5 μm paraffin sections were prepared using standard procedures. For the immunohistochemical assays, the sections were sealed in 5% w/v bovine serum albumin for 30 min at room temperature and then incubated with primary antibodies at 4°C overnight. The primary antibodies are listed in Supplementary Table S2. Non-immune IgG was substituted for the primary antibody as the negative control. On the following day, the slides were then stained with the corresponding secondary antibodies followed by diaminobenzidine (Agilent Technologies, Santa Clara, CA, United States) and hematoxylin counterstaining.

Images were recorded under the microscope BX53F (Olympus, Tokyo, Japan). H-score was performed to compare the expression of CDYL in IHC staining. H score = Σpi(i + 1), where i was the intensity of staining (range from 0 for negative staining to 3 for the most intense staining), and Pi was the percentage of cells stained at each intensity (0–100%).

### Cell Culture

The human EEC line Ishikawa was obtained from European Collection of Authenticated Cell Cultures (ECACC), and were authenticated by DNA profiling using short tandem repeat (STR) analysis (Supplementary Figure S1A) and mycoplasma free (Supplementary Figure S1B). Isolation of primary human EECs and ESCs was performed as previously described (Xu et al., 2018)^.^ Briefly, endometrial biopsy samples were processed within 4 h of collection. The tissue was rinsed with PBS and minced into small pieces, then were digested with 1 mg/mL collagenase type I (Thermo Fisher Scientific) in a 37°C incubator with shaking for 30 min. The mixture was then passed through two 100 and 40 μm sieves successively (Millipore). The epithelial cells were hold by the 40 μm sieve and the filtrate contained stromal cells and a few epithelial cells. Then the 40 μm sieve was washed with PBS to collect the epithelial cells. Primary EECs or ESCs were pelleted, respectively, by centrifuged at 100 *g* for 5 min. Epithelial cells were plated into the collagen coated culture flask. The immunofluorescence staining of Cytokeratin 7 was performed on the cultured primary EECs for identification (Supplementary Figure S5). For stromal cells culture, the medium was refreshed 30 min later to remove the epithelial cell contamination. Ishikawa cells, primary human EECs, and ESCs were cultured in DMEM/F12 medium (Thermo Fisher Scientific). All cell medium was supplemented with 10% fetal bovine serum (FBS) and 1% penicillin-streptomycin. Cells were cultured at 37°C under 5% CO_2_ in humidified air, and harvested by brief incubation in 0.25% (w/v) Trypsin-EDTA (Thermo Fisher Scientific). The medium was renewed every 2–3 days.

### ShRNA Knockdown and Plasmid Over-Expression of *CDYL*

Short hairpin RNA (shRNA) against human *CDYL* and negative control shRNA were purchased from Hanbio (Shanghai, China), the targeting sequence of *CDYL* was cloned into (pHB-U6-MCS-CMV-ZsGreen-PGK-PURO) plasmid, while *CDYL* over-expression and mock plasmids were purchased from FulenGen (Guangzhou, China). Cells were transfected with shRNAs or plasmids according to the manufacturer’s instructions. Briefly, for the *CDYL* knockdown assay, the Ishikawa cells, primary EECs, and ESCs were seeded at 30% confluence 24 h prior to shRNAs transfection in 60 mm dishes, and were incubated with 2 × 10^7^ TU/mL shRNAs, along with 10 μg/mL polybrene. The transduced cells were selected with puromycin (1 μg/mL) after 3 days of transduction. For the over-expression assay, cells were seeded at 70% confluence 24 h prior to over-expression plasmids transfection in 6-well plates; 1 μg over-expression plasmids, 4 μL X-tremeGENE 9 DNA transfection reagent (Roche), and 200 μL Opti-MEM (Thermo Fisher Scientific) were mixed and incubated for 15 min at room temperature. Cells were then transfected by the mixture and incubated for 72 h. The knockdown and over-expression efficiency was determined with RT-qPCR and Western blot. The targeting sequences of *CDYL* shRNA used in this study are listed in Supplementary Table S3.

### Whole-Genome Expression Profile Analysis

Total RNA were extracted from the negative control and *CDYL*-knockdown Ishikawa cells using the TRIzol^®^ Reagent according the manufacturer’s instructions (Thermo Fisher Scientific), and genomic DNA were removed using DNase I (TaKara). RNA-seq transcriptome library was prepared following TruSeq^TM^ RNA sample preparation Kit from Illumina (San Diego, CA, United States). The paired-end RNA-seq sequencing library was sequenced with the Illumina HiSeq 4000 (2 × 150 bp read length). After quality control, the clean reads were separately aligned to reference genome (GRCh38.p10) with orientation mode using TopHat software^1^ (version 2.0.0). Then principle components analysis (PCA), differential expression gene (DEG) analysis, and functional enrichment analysis were carried out according to the previously published methods (Guo et al., 2018) (GSE141239). The protein–protein interaction (PPI) network of selected DEGs was constructed by String and Cytoscape online tools. The interaction between marker genes of endometrial receptivity was constructed based on the published references using String^2^ and Cytoscape 3.6.1 (National Resource for Network Biology).

### Cell Migration Assay

For the wound healing experiment, Ishikawa cells and ESCs (1 × 10^6^ cells) with or without treatment were plated onto six-well plates for 24 h to reach sub-confluence. Then, the cell surface was scratched with a pipet tip to form a cell-free gap. Cells were rinsed carefully with PBS and cultured in serum deprivation media (DMEM/F12 medium with 0.1% FBS). The percentage of closure was measured at 0, 24, and 48 h, respectively. Images were analyzed with the Image J 1.46r (Nunes and Dias, 2017).

Transwell migration assay was performed as previously described (Chen et al., 2018). Briefly, 5 × 10^4^ Ishikawa cells, EECs, and ESCs cells were suspended in 100 μL serum-free DMEM/F12 medium and seeded into the upper chamber of each insert. Then, 600 μL of DMEM containing 15% FBS was added to a 24-well plate. After incubation for 48 h at 37°C in 5% CO_2_, cells that had migrated were stained with crystal violet and then counted under optical microscope. Two observers counted by hand independently. Each experiment was repeated three times.

### Trophoblast Outgrowth Analysis

Endometrial stromal cells were isolated and cultured from four biopsies of controls, as described above. ESCs transfected with *CDYL* shRNA or negative shRNA were grown to confluence in a 24-well plate. Cells were then decidualized by treatment with 10 nM β-estradiol (E2758, Thermo Fisher Scientific), 1 μM progesterone (V900699, Thermo Fisher Scientific), and 1 mM 8-Br-cAMP (ab141448, Abcam) for 48 h. Then hatched blastocysts from C57BL/6 mice with a normal morphology were cocultured with confluent monolayers of decidualized ESCs for 72 h in DMEM/F12 complete medium. The morphological images were captured by microscope TS2 (Nikon, Tokyo, Japan). The trophoblast outgrowth areas were outlined manually and measured using Image J 1.46r.

### Statistical Analysis

Means of every group were compared by two-tailed Student’s *t*-test with unequal variance assumed. Differences between more than two groups were compared using one-way analysis of variance if normality (and homogeneity of variance) assumptions are satisfied. Multiple comparisons were analyzed based on Bonferroni test. Otherwise, the Kruskal–Wallis test followed by Mann–Whitney *U*-test was performed to analyze the data. Threshold values, sensitivity, and specificity were calculated using receiver operating characteristic (ROC) curves. All statistical analysis was performed using the SPSS 23.0 (IBM, United States) and statistical significance was defined as *P* < 0.05.

## Results

### Demographic Characteristics

The demographic characteristics of the recruited control group and RIF group are summarized in Table 1. Except for the times of embryos transfers, there was no significant difference between these two groups (*P* > 0.05). Similarly, no significant difference among the two different menstrual phase groups, according to the demographic characteristics listed in Table 2.

### *CDYL* Was Upregulated in the Endometrium of Mid-Secretory Phase but Decreased in RIF Cases

**FIGURE 1:**
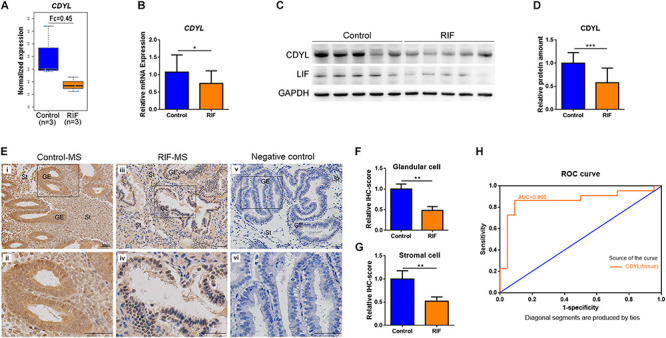
The expression of *CDYL* was significantly decreased in the endometrium of women with RIF. **(A)** The expression levels of *CDYL* in the control (*n* = 3) and RIF (*n* = 3) groups using microarray assay. **(B)** The mRNA levels of *CDYL* in the endometrium of the control (*n* = 22) and RIF (*n* = 22) groups. **(C)** Representative western blot analysis of CDYL and LIF in the control and RIF groups. **(D)** Densitometric quantification of CDYL in the endometrium of the control (*n* = 22) and RIF (*n* = 22) groups. **(E)** Immunohistochemistry staining and **(F,G)** semi-quantification of CDYL protein expression in the control (*n* = 5) and RIF patients (*n* = 5). Bar = 50 μm. **(H)** Receiver operating characteristic (ROC) curve plotting of the true positive vs. false positive rate, and the optimal cutoff value for endometrial issue for CDYL measurements. All data are presented as mean ± SD. * *P* < 0.05; ** *P* < 0.01; *** *P* < 0.001.

Our previous microarray assay found that there were 2519 differentially expressed genes (DEGs) identified in the RIF group, with a fold change of > 2 and an adjusted *P*-value of < 0.05 (NCBI GSE103465) (Guo et al., 2018). Among these DEGs, *CDYL* was significantly decreased in the RIF group (Figure 1A). In the present study, this finding was confirmed by RT-qPCR and Western blot analyses. As shown in Figure 1B, the mRNA level of *CDYL* dropped in the RIF group (*P* < 0.05). Similarly, the protein level of CDYL was much lower in women with RIF (*P* < 0.001) (Figures 1C,D and Supplementary Figure S4), consistent with the protein level of the endometrial receptivity marker LIF (Figure 1C).

We then executed the immunostaining in aim to investigate the cellular localization of CDYL in the human endometrial tissue. The results showed that CDYL was strongly expressed in both the epithelial and stromal cells at the mid-secretory phase in the control group (Figures 1Ei,ii). However, the signal of CDYL was dramatically reduced in the endometrial cells of women with RIF (*P* < 0.01) (Figures 1Eiii,iv,F,G). Using ROC curve analysis, we further verified a potential functional role of CDYL in evaluating endometrial receptivity, as the ROC area under the curve (AUC) was 0.866 [0.744–0.988] for tissue CDYL measurements (Figure 1H).

**FIGURE 2:**
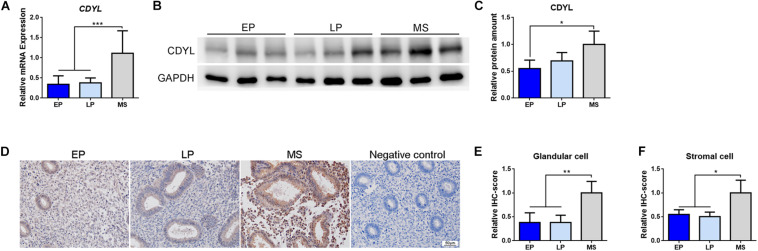
The expression of *CDYL* was increased in the endometrium during the menstrual cycle. **(A)** RT-qPCR, **(B)** Western blot analysis, and **(C)** densitometric quantification of CDYL expression in endometrium during the menstrual cycle. EP, early proliferative phase, *n* = 8; LP, late proliferative phase, *n* = 12; MS, mid-secretory phase, *n* = 22. **(D)** Immunohistochemistry staining and **(E,F)** semi-quantification of CDYL protein expression in endometrial cells during the normal menstrual cycle. EP, *n* = 5; LP, *n* = 5; MS, *n* = 5. All data are presented as mean ± SD. * *P* < 0.05; ** *P* < 0.01; *** *P* < 0.001.

We then measured the expression of *CDYL* mRNA and protein in the endometrial tissues during the normal menstrual cycle. As shown in Figure 2, the mRNA and protein levels of *CDYL* increased in the endometrium from the early proliferative phase to mid-secretory phase, with a peak level in the mid-secretory phase. These results suggested that CDYL might be involved in the regulation of the endometrial receptivity.

### Knockdown of *CDYL* Affected the Migration Ability and Transcriptome Profile in Ishikawa Cells

**FIGURE 3:**
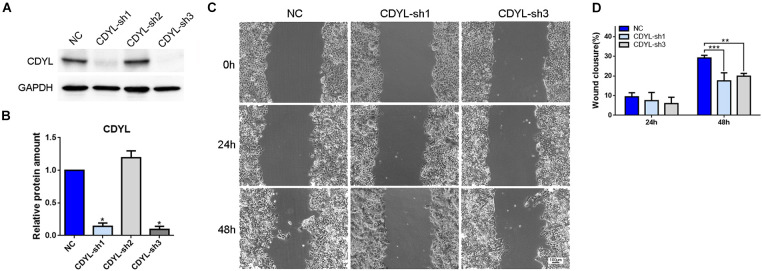
*CDYL* knockdown suppressed the cell migration capability in Ishikawa cells. The CDYL-knockdown efficiency was shown by Western blot analysis **(A)** and densitometric quantification **(B)**. **(C)** Wound healing assay was used to examine the effect of *CDYL* silencing on cell migration. **(D)** The histograms show the quantitated percentage of wound closure. Bar = 100 μm. *N* = 3. All data are presented as mean ± SD. * *P* < 0.05; ** *P* < 0.01; *** *P* < 0.001.

Based on the above findings, we suppressed the expression of *CDYL* by shRNA approach in human endometrial cell line Ishikawa (Figures 3A,B). The inhibition efficiency was 83.45 ± 5.07% by *CDYL*-sh1 and 88.51 ± 4.64% by *CDYL*-sh3. As a consequence, the cell migration ability was inhibited in *CDYL*-sh Ishikawa cells (*P* < 0.01) (Figures 3C,D).

**FIGURE 4:**
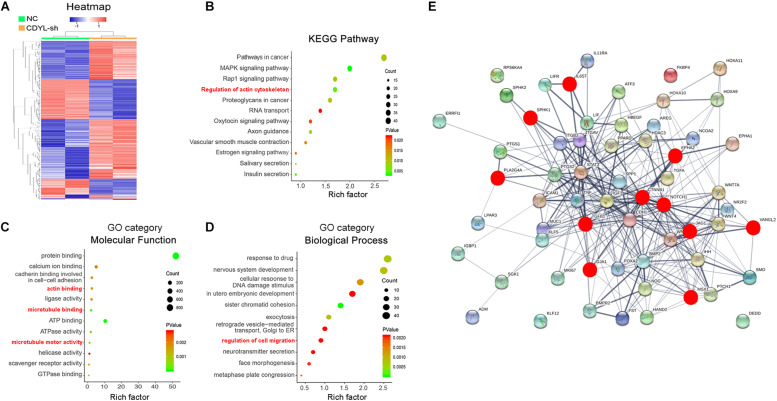
Analysis of transcriptome alternation in *CDYL* knockdown Ishikawa cells. **(A)** Hierarchical clustering of differentially expressed genes (DEGs). Rows in the heatmap correspond to DEGs, and columns correspond to individual samples. Functional enrichment analysis of DEGs, including KEGG pathway **(B)**, GO category molecular function **(C)**, and biological process **(D)**. **(E)** Protein–protein interaction (PPI) network analysis of the top 56 genes involved in the endometrial receptivity and implantation. Genes affected by the CDYL knockdown are marked in red.

Therefore, we compared the transcriptome between the *CDYL*-sh Ishikawa cells and their control counterparts using high-throughput RNA sequencing (RNA-seq) (*n* = 2 each) (GSE141239). We identified 1738 DEGs in the *CDYL*-sh group (Figure 4A). Interestingly, we noticed a class of DEGs enriched in the clusters of cytoskeleton and migration regulation, as shown in the Kyoto Encyclopedia of Genes and Genomes (KEGG) and Gene Ontology (GO) analyses based on their biological processes and molecular functions (Figures 4B–D, terms in red). Using String and Cytospace online tools, genes in these clusters were mapped into an interaction network (Figure 4E and Supplementary Figure S2). Strikingly, we found genes including catenin beta 1 (*CTNNB1*), *NOTCH1* (notch receptor 1), and *GJA1* (gap junction protein, alpha), which are well-known regulators of endometrial receptivity (Zhang et al., 2012; Van Sinderen et al., 2014; Winterhager and Kidder, 2015; Su et al., 2016; Yu et al., 2017; Zheng et al., 2017), centered in this network. In addition, transcripts of *EPHA2* (EPH Receptor A2), *IL6ST* (Interleukin 6 Signal Transducer), *JAG1* (Jagged canonical notch ligand 1), *MSX1* (msh homeobox 1), *PLA2G4A* (phospholipase A2, group IVA), *TGFB1* (transforming growth factor beta 1), *SPHK1* (sphingosine kinase 1), and *VANGL2* (VANGL planar cell polarity protein 2) exhibited > 1.5-fold change of expression (red dot), which have been proved functional in the endometrial receptivity regulation. Importantly, *CTNNB1* was identified as the most important hub gene in the PPI network construction.

### Knockdown of *CDYL* Decreased the *CTNNB1* Expression in Ishikawa Cells, Primary EECs, and Primary ESCs

**FIGURE 5:**
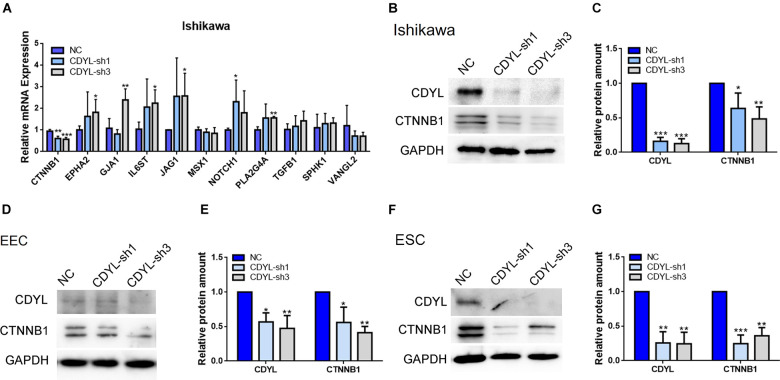
The decreased expression of *CTNNB1* after *CDYL* knockdown in Ishikawa cells, EECs, and ESCs. **(A)** DEGs involved in the endometrial receptivity and implantation were validated by RT-qPCR. Protein expression levels of CDYL and CTNNB1 were decreased after CDYL silencing in Ishikawa cells **(B,C)**, EECs **(D,E)**, and ESCs **(F,G)** shown by Western blot and densitometric quantification. *N* ≥ 3. All data are presented as mean ± SD. * *P* < 0.05; ** *P* < 0.01; *** *P* < 0.001.

We then validated the RNA-seq results of selected DEGs by RT-qPCR (Figure 5A). Among these genes, the mRNA level of *CTNNB1* was clearly decreased in *CDYL*-sh Ishikawa cells. Using shRNA technique, we convincingly verified that, in Ishikawa cells, primary EECs, and primary ESCs, the expression of CTNNB1 isoforms was reduced responding to the *CDYL* knockdown treatment (*P* < 0.05) (Figures 5B–G and Supplementary Figure S6). These findings suggested that the expression of *CTNNB1* in endometrial cells was regulated by *CDYL*.

### *CTNNB1* Was Decreased in the Endometrium of Women With RIF

**FIGURE 6:**
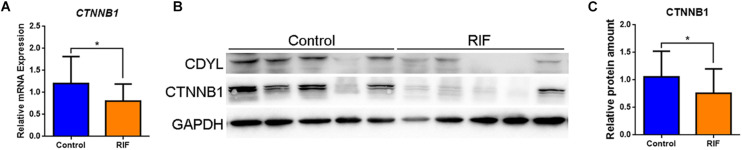
The expression of *CTNNB1* was decreased in the endometrium of women with RIF. **(A)** The mRNA expression levels of *CTNNB1* in control (*n* = 22) and RIF (*n* = 22) groups. **(B,C)** The protein expression levels of CTNNB1 in the control (*n* = 22) and RIF (*n* = 22) groups. All data are presented as mean ± SD. * *P* < 0.05.

Therefore, we measured the expression levels of *CTNNB1* mRNA and protein in the endometrial tissues from the control and RIF groups. Our results showed that the expression of *CTNNB1* was down-regulated in the endometrium of the RIF group as well (*P* < 0.05) (Figures 6A–C).

### *CDYL*-Mediated *CTNNB1* Expression Was Involved in the Regulation of Cell Migration in Endometrial Cells

**FIGURE 7:**
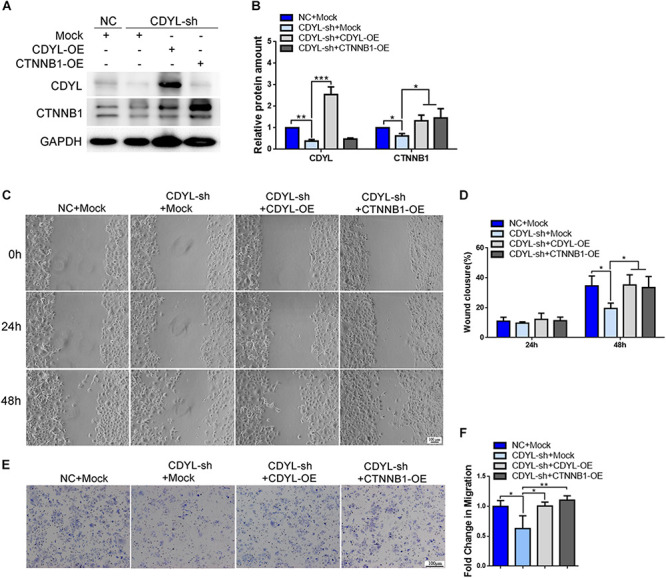
Effects of *CDYL* knockdown/over-expression on CTNNB1 expression and cell migration in Ishikawa cells. **(A,B)** Effects of CDYL on CTNNB1 expression level in Ishikawa cells by Western blot analysis (*n* = 3). Effects of CDYL on cell migration in Ishikawa cells by wound-healing assay **(C,D)** and transwell migration assay **(E,F)**. Bar = 100 μm. N = 3. All data are presented as mean ± SD. * *P* < 0.05; ** *P* < 0.01; *** *P* < 0.001.

Using both knockdown- and overexpression-based approaches, we confirmed the positive regulatory role of *CDYL* in the expression of *CTNNB1* in Ishikawa cells (Figures 7A,B and Supplementary Figure S3). Notably, the migration capability was damaged in *CDYL*-sh Ishikawa cells (*P* < 0.05), but restored after transfection with either *CDYL*- or *CTNNB1*-overexpression plasmids (Figures 7C–F).

**FIGURE 8:**
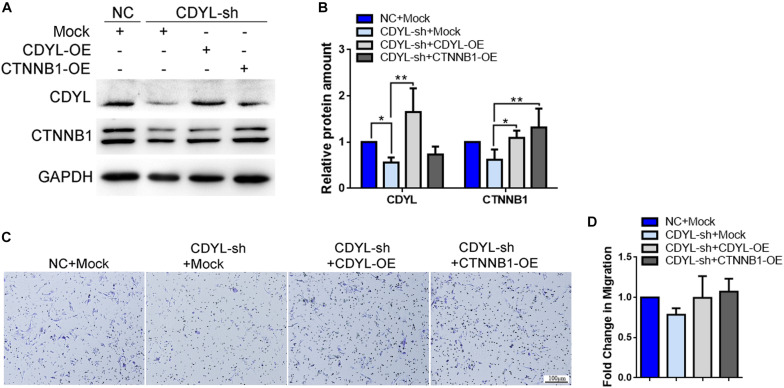
Effects of *CDYL* knockdown/over-expression on the expression of CTNNB1 and cell migration in primary endometrial epithelial cells. **(A,B)** Effects of CDYL on CTNNB1 expression level in EECs by Western blot analysis. **(C,D)** Effects of CDYL on cell migration in EECs by transwell migration assay. Bar = 100 μm. *N* = 3. All data are presented as mean ± SD. * *P* < 0.05; ** *P* < 0.01.

**FIGURE 9:**
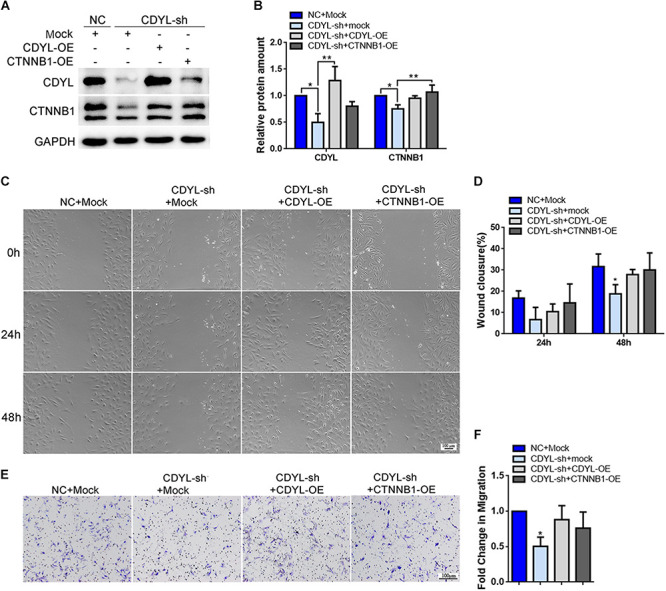
Effects of *CDYL* knockdown/over-expression on the expression of CTNNB1 and cell migration in primary endometrial stromal cells. **(A,B)** Effects of CDYL on CTNNB1 expression level in ESCs by Western blot analysis. Effects of CDYL on cell migration in ESCs by wound-healing assay **(C,D)** and transwell migration assay **(E,F)**. Bar = 100 μm. *N* = 3. All data are presented as mean ± SD. * *P* < 0.05; ** *P* < 0.01.

Similarly, the expression of *CTNNB1* was significantly decreased after knockdown of *CDYL*, which could be reversed by over-expression of either *CDYL* or *CTNNB1* in both primary EECs and ESCs (Figures 8A,B, 9A,B, respectively). The results from the transwell assay demonstrated that there was an inclination of reduced migration capability of EECs after *CDYL*-knockdown (Figures 8C,D). In contrast, the migration capability of ESCs was visibly impaired after *CDYL*-knockdown, as shown in the transwell assay and the wound healing experiment (*P* < 0.05). As expected, the declined migration rate in *CDYL*-sh ESCs could be partially rescued by over-expression of either *CDYL* or *CTNNB1* (Figures 9C–F and Supplementary Figure S7).

### *CDYL* Knockdown in ESCs Inhibited the Trophoblast Outgrowth *in vitro*

**FIGURE 10:**
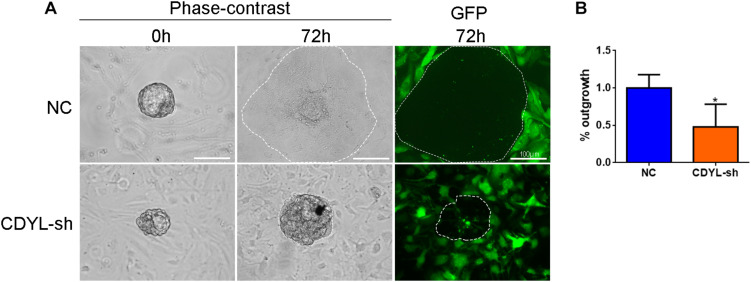
*CDYL* knockdown in ESCs inhibited the trophoblast outgrowth *in vitro.* **(A)** Representative images showing the spreading mouse trophoblast (dotted line) that co-cultured on control or *CDYL*-sh ESCs. **(B)** Quantification of the area of outgrowth. *N* = 4, mean value of control group was set to 100%. Data are presented as mean ± SD. * *P* < 0.05.

We further extended our observations on a heterologous co-culture system of embryo implantation. Human ESCs were transfected with *CDYL* shRNA and decidualized, mouse blastocysts were then plated onto treated ESCs. After 72 h, *CDYL*-sh ESCs displayed a marked deficiency in supporting trophoblast outgrowth, which mimic the process of trophoblast invasion during implantation *in vivo* (Figure 10).

## Discussion

Even though a rapid progress of assisted reproductive technology (ART) has been achieved since 21st century, there are still a number of infertile women experiencing frequent ART failure after repeated attempts (Kushnir et al., 2017). Indeed, RIF with impaired endometrial receptivity is still a great challenge for clinicians toward improving pregnancy outcomes in ART. Despite intensive research efforts over several decades, the physiological and pathological mechanisms underlying RIF remain elusive (Zeyneloglu and Onalan, 2014).

Endometrium achieves the cyclical change involving regeneration, proliferation, differentiation, and desquamation in the reproductive age controlled by the ovarian steroidal hormones (Gellersen and Brosens, 2014). More and more evidence supported that these dynamic morphological and functional changes during the menstrual cycles are epigenetically regulated (Grimaldi et al., 2011; Tamura et al., 2014; Katoh et al., 2018). Epigenetic modifications mainly refer to the DNA methylation and histone modifications, of which histone methylation and acetylation are most studied (Zhou et al., 2011), that aberrant epigenetic modifications may be associated with the progress of endometriosis (Izawa et al., 2013; Forte et al., 2014; Zhang et al., 2017), recurrent miscarriage (Arias-Sosa et al., 2018; Yu et al., 2018), and implantation failure. For instance, *HDAC3* is critical for endometrial receptivity, such that loss of *HDAC3* caused an impaired decidualization through the abnormal activation of *COL1A1/2* genes in humans (Kim and Yoo, 2019).

CDYL, the CDYL protein, is a novel epigenetic regulator that comprised of a C-terminal crotonase-like fold and an N-terminal chomodomain (Liu S. et al., 2017). Based on the properties of chromodomain, CDYL is known as an H3K9me3 and H3K27me3 reader, which plays an essential role in the transmission/restoration of histone markers. Additionally, CDYL functions in the maintenance of cell identity by regulating the transcriptional profile epigenetically (Liu Y. et al., 2017; Qiu et al., 2019). CDYL has been proved participating in several physiological activities, including neuronal migration, neural development, transformation of tumor cells, even the X chromosome inactivation (Escamilla-Del-Arenal et al., 2013; Wu et al., 2013; Qin et al., 2017). Importantly, CDYL/Cdyl is a critical element for mouse spermatogenesis and male fertility (Xia et al., 2019). In the present study, we demonstrated for the first time that the expression level of CDYL was decreased in the mid-secretory phase of endometrium in women with RIF, which probably damaged the endometrial receptivity by affecting the migration of endometrial cells.

Using gene expression microarray analysis, we discovered the downregulation of *CDYL* mRNA in the endometrial samples obtained from patients with RIF (Figure 1) (Guo et al., 2018). We further detected the significantly lower expression of CDYL protein in the RIF group, consistent with the expression of LIF (Figure 1C), which is a marker of endometrial receptivity (Paiva et al., 2009; Cheng et al., 2017; Vagnini et al., 2019). In addition, the results obtained from ROC analysis revealed the high sensitivity and specificity to discriminate endometrial receptivity based on the CDYL expression levels of the endometrial tissues. It brought out an interesting question whether the high expression of CDYL in the mid-secretory phase is necessary for the establishment of endometrial receptivity. In another word, if the decreased expression of CDYL contributes to the impaired endometrial receptivity in women with RIF.

As discussed in Section “Introduction,” it has been well acknowledged that the migration capability of endometrial epithelial and stromal cells is essential for the later implantation process (Gonzalez et al., 2011; Weimar et al., 2013; Xu et al., 2019). Using the shRNA approach, we found the knockdown of *CDYL* depressed the migration ability of Ishikawa cells (Figure 3) and primary endometrial cells (Figures 8, 9). We have further proved that *CDYL*-sh ESCs displayed a marked deficiency in supporting trophoblast outgrowth when compared to the control cells (Figure 10), which suggesting the impaired capacity of decidualization.

In order to investigate the downstream effectors of CDYL that affecting cell migration, we examined the transcriptional profile in Ishikawa cells after shRNA-mediated knockdown of *CDYL*. We identified 1738 DEGs, among which, *CTNNB1* is evolutionarily conserved and plays an important role in the process of pregnancy. Animal studies using mouse models have shown that CTNNB1 is required for proper development of uterine functions, including estrous cycle establishment, embryo implantation, and placental vascularization, while the targeted depletion of *CTNNB1* in uterus results in impaired decidualization and infertility (Mohamed et al., 2005; Jeong et al., 2009; Zhang et al., 2012).

In the present study, we have detected the deceased expression of *CTNNB1* in the endometrium from RIF cases (Figure 6). Using both shRNA and overexpression approaches, we have verified the expression of *CTNNB1* was positively regulated by *CDYL in vitro* (Figures 5, 7–9). As we expected, the inhibitory effects of *CDYL* knockdown on cell migration could be efficiently rescued by *CTNNB1* overexpression (Figures 7, 9). Combining all our findings, we supposed that *CDYL* functions in the regulation of endometrial cell migration via mediating the *CTNNB1* expression. In that case, the underexpression of *CDYL* in endometrium could be a risk factor of poor endometrial receptivity and RIF. It should be noted that the precise mechanism needs to be carefully investigated, given the *CDYL* knockdown inducing profound alteration of transcriptome (Figure 4) and epigenetic modifications (Supplementary Figures S8, S9). These attempts would deepen our understanding of the molecular mechanisms underlying RIF, which may pave the way toward new diagnostic and therapeutic strategies.

## Data Availability Statement

The datasets generated for this study can be found in the GSE141239.

## Ethics Statement

The studies involving human participants were reviewed and approved by the Institutional Ethics Committee of Ruijin Hospital, Shanghai Jiao Tong University, School of Medicine. The patients/participants provided their written informed consent to participate in this study. The animal study was reviewed and approved by the Animal Ethics Committee of Shanghai Jiao Tong University School of Medicine (DAS-A-2016-038).

## Author Contributions

AZ, BX, and XX designed the experiments. BX, DZ, and XJ recruited the participants and collected the samples. XZ performed the experiments. XZ and XX analyzed the data and wrote the manuscript. H-MC and PL reviewed the manuscript for intellectual content. All authors read and approved the final version of the manuscript.

## Conflict of Interest

The authors declare that the research was conducted in the absence of any commercial or financial relationships that could be construed as a potential conflict of interest.

## Footnotes

1
http://tophat.cbcb.umd.edu/

2
https://string-db.org/cgi/input.pl

## Supplementary Material

FIGURE S1 Characterization of human endometrial epithelial cell line Ishikawa. **(A)** DNA profiling using short tandem repeat (STR) analysis. **(B)** DAPI staining for mycoplasma-free test.

FIGURE S2 Protein–protein interaction (PPI) network analysis of the DEGs involved in the cell motility regulation.

FIGURE S3 Induced expression of CTNNB1 by *CDYL* overexpression in **(A)** Ishikawa, **(B)** EECs, and **(C)** ESCs.

FIGURE S4 Western blot detection for CDYL in control and RIF samples shown in Figure 1C.

FIGURE S5 Morphology and Cytokeratin 7 staining of the cultured primary EECs.

FIGURE S6 The differential expression of CTNNB1 isoforms in Ishikawa, EECs, and ESCs.

FIGURE S7 Effects of *CDYL* knockdown/over-expression on the expression of CTNNB1 and cell migration in primary endometrial stromal cells from RIF patients. **(A)** Effects of CDYL on CTNNB1 expression level in EECs by Western blot analysis. **(B)** Effects of CDYL on cell migration in ESCs by wound-healing assay. Bar = 100 μm.

FIGURE S8 Detection of critical histone modifications in *CDYL*-sh Ishikawa cells.

FIGURE S9 Detection of critical histone modifications in endometrial samples from control and RIF patients. **(A)** Representative results from western blot analysis. **(B)** Quantification of detected histone modifications in both control (*n* = 16) and RIF (*n* = 16) groups.

TABLE S1 Primers used in this study.

TABLE S2 Antibodies used in this study.

TABLE S3 The effective target sequences for CDYL shRNA.
