# Correction: Loss of CDYL results in suppression of CTNNB1 and decreased endometrial receptivity

**DOI:** 10.3389/fcell.2025.1605628

**Published:** 2025-06-25

**Authors:** Xiaowei Zhou, Bufang Xu, Dan Zhang, Xiaoping Jiang, Hsun-Ming Chang, Peter C. K. Leung, Xiaoyu Xia, Aijun Zhang

**Affiliations:** ^1^ Department of Reproductive Medical Center, Ruijin Hospital, School of Medicine, Shanghai Jiao Tong University, Shanghai, China; ^2^ Department of Obstetrics and Gynecology, Chinese People’s Armed Police Force Shanghai Corps Hospital, Shanghai, China; ^3^ Department of Obstetrics and Gynaecology, BC Children’s Hospital Research Institute, The University of British Columbia, Vancouver, BC, Canada; ^4^ Department of Histoembryology, Genetics and Developmental Biology, School of Medicine, Shanghai Jiao Tong University, Shanghai, China; ^5^ Shanghai Key Laboratory of Reproductive Medicine, Shanghai Jiao Tong University, Shanghai, China

**Keywords:** recurrent implantation failure, chromodomain Y like, catenin beta 1, migration, endometrial receptivity

In the published article, there was an error in Figure 3C. The authors have identified that the image for the CDYL-sh1 48h treatment was inadvertently duplicated from the CDYL-sh3 24h treatment due to an error during figure preparation. The corrected Figure 3C and its caption appear below.

**FIGURE 3:**
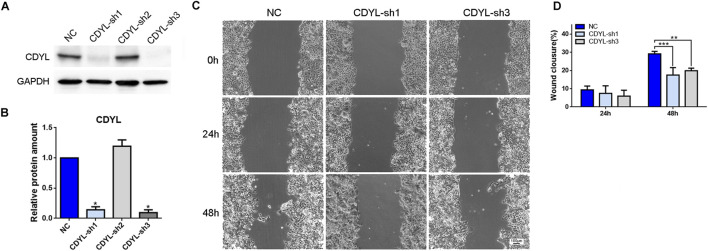
CDYL knockdown suppressed the cell migration capability in Ishikawa cells. The CDYL-knockdown efficiency was shown by Western blot analysis **(A)** and densitometric quantification **(B)**. **(C)** Wound healing assay was used to examine the effect of CDYL silencing on cell migration. **(D)** The histograms show the quantitated percentage of wound closure. Bar = 100 µm. N = 3. All data are presented as mean ± SD. *P < 0.05; **P < 0.01; ***P < 0.001.

In the published article, there was an error in Figure 5F. Although the Western blot for CDYL in Figure 5F was performed on the same experimental day, it was not run on the same membrane as CTNNB1 and GAPDH, due to an error during figure preparation. The corrected Figure 5F and its caption appear below.

**FIGURE 5:**
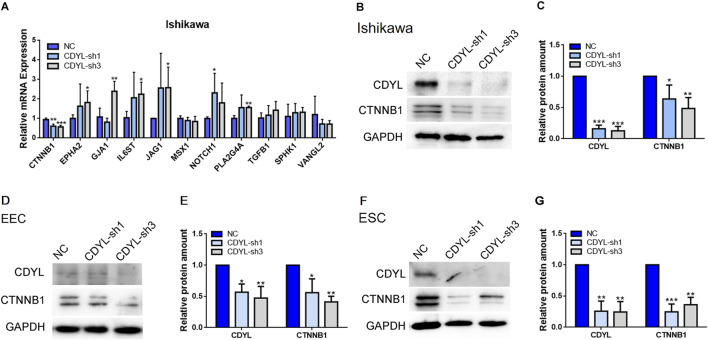
The decreased expression of CTNNB1 after CDYL knockdown in Ishikawa cells, EECs, and ESCs. **(A)** DEGs involved in the endometrial receptivity and implantation were validated by RT-qPCR. Protein expression levels of CDYL and CTNNB1 were decreased after CDYL silencing in Ishikawa cells **(B,C)**, EECs **(D,E)**, and ESCs **(F,G)** shown by Western blot and densitometric quantification. N ≥ 3. All data are presented as mean ± SD. *P < 0.05; **P < 0.01; ***P < 0.001.

The authors apologize for these errors and state that this does not change the scientific conclusions of the article in any way. The original article has been updated.

