# Supplementary figures and images for: Loss of CDYL Results in Suppression of CTNNB1 and Decreased Endometrial Receptivity
Source: Front Cell Dev Biol. 2020 Feb 25;8:105. doi: 10.3389/fcell.2020.00105 (PMC7051920; doi:10.3389/fcell.2020.00105)

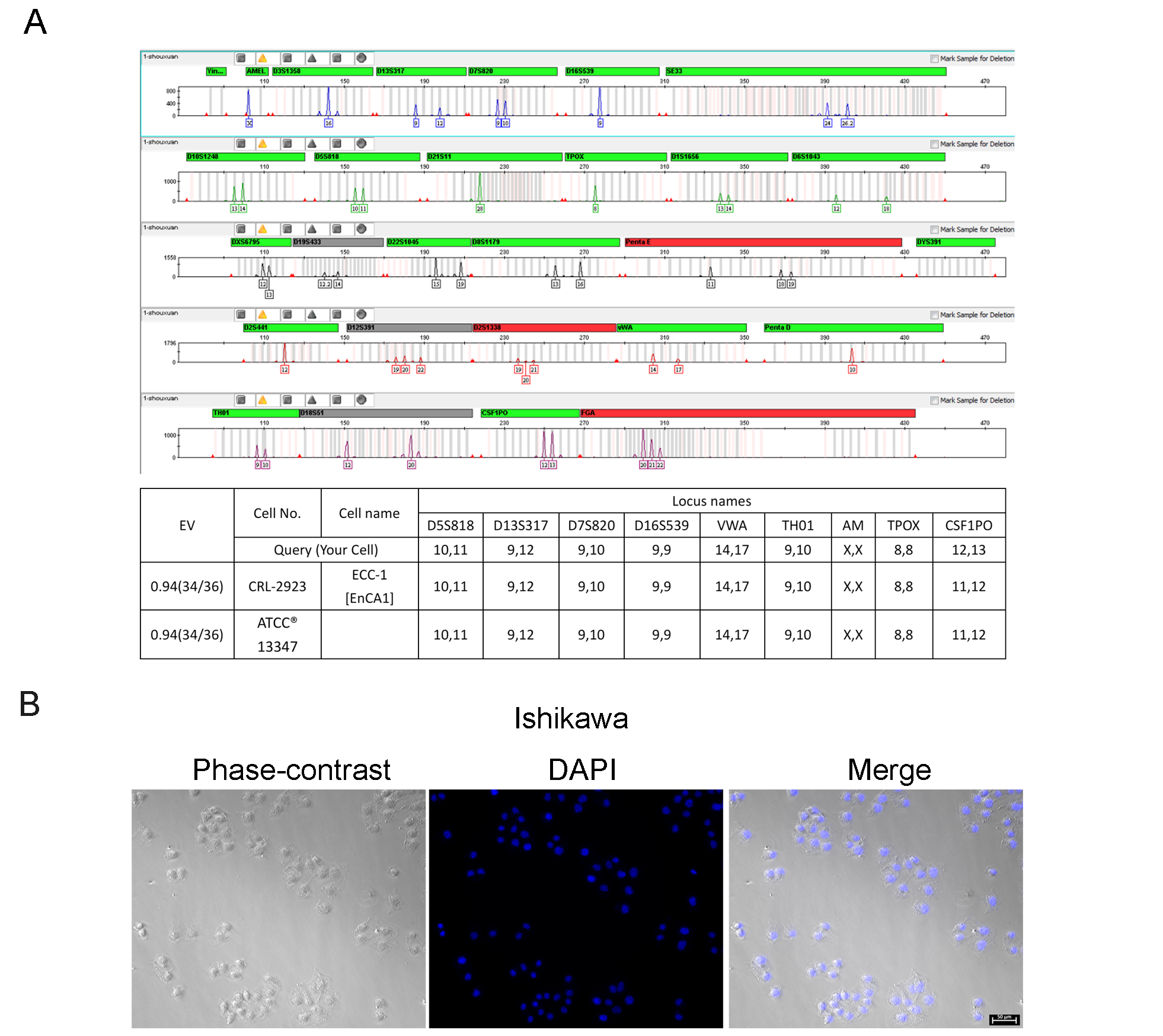

Supplement: FIGURE S1 — Characterization of human endometrial epithelial cell line Ishikawa. (A) DNA profiling using short tandem repeat (STR) analysis. (B) DAPI staining for mycoplasma-free test. [file Image_1.TIF]

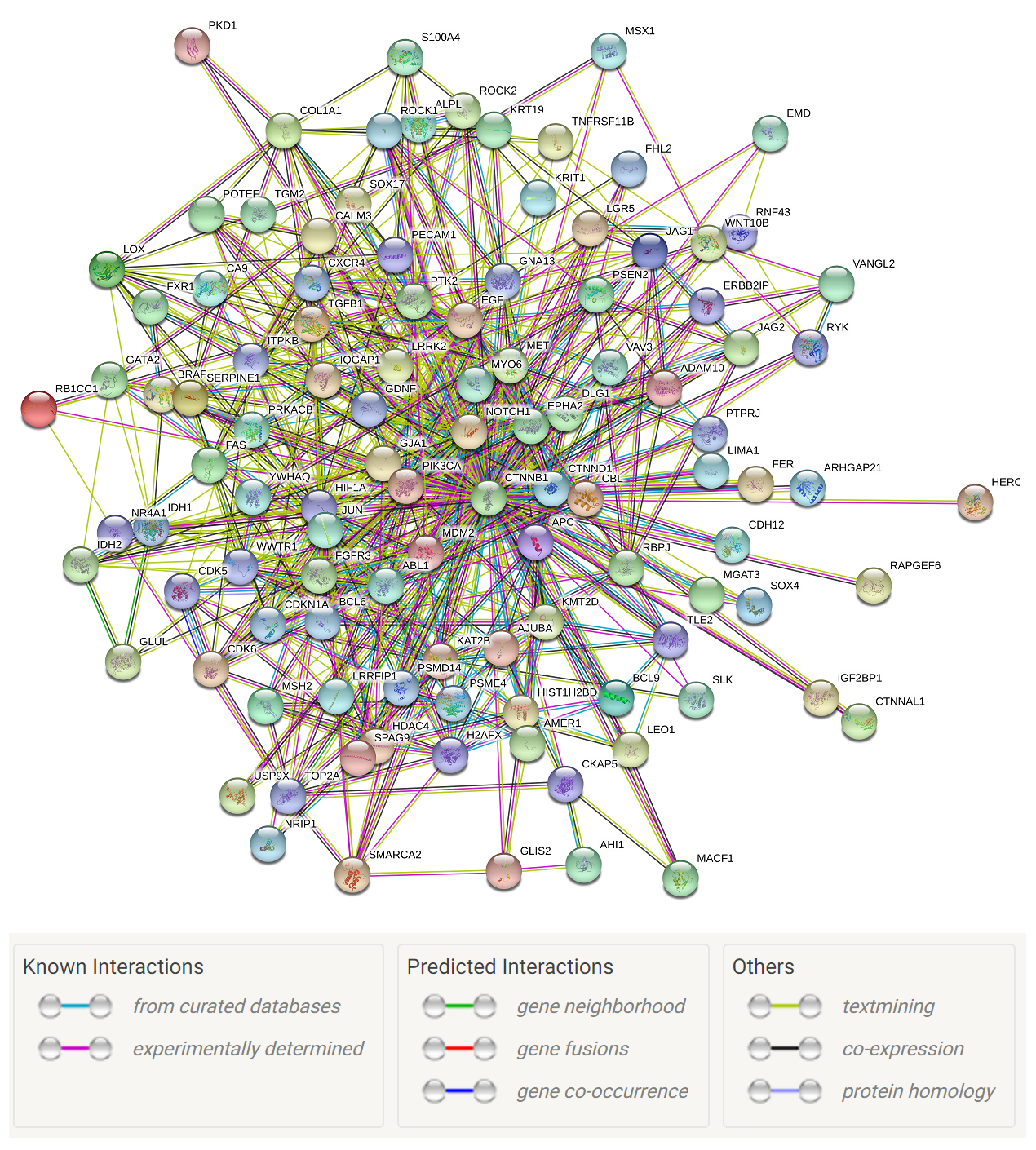

Supplement: FIGURE S2 — Protein–protein interaction (PPI) network analysis of the DEGs involved in the cell motility regulation. [file Image_2.TIF]

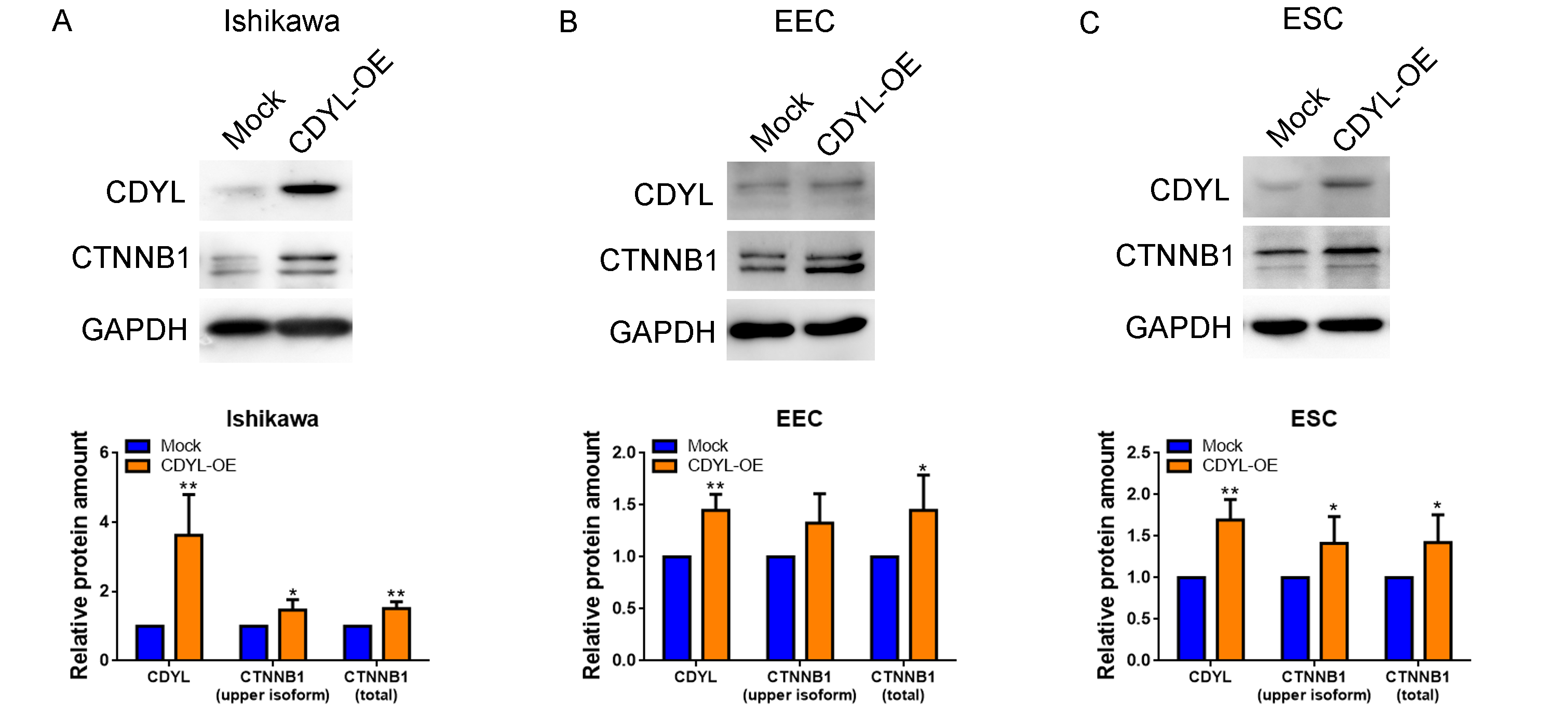

Supplement: FIGURE S3 — Induced expression of CTNNB1 by CDYL overexpression in (A) Ishikawa, (B) EECs, and (C) ESCs. [file Image_3.TIF]

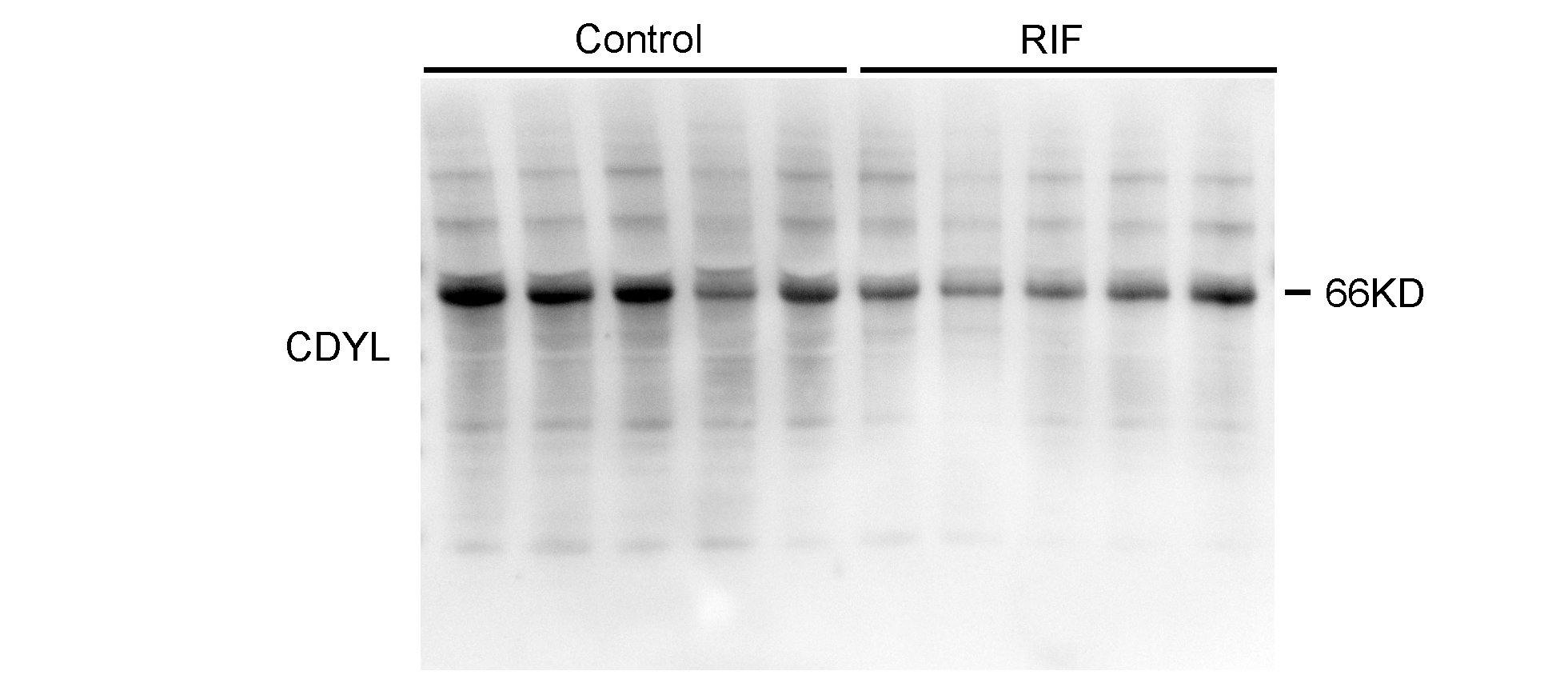

Supplement: FIGURE S4 — Western blot detection for CDYL in control and RIF samples shown in Figure 1C. [file Image_4.TIF]

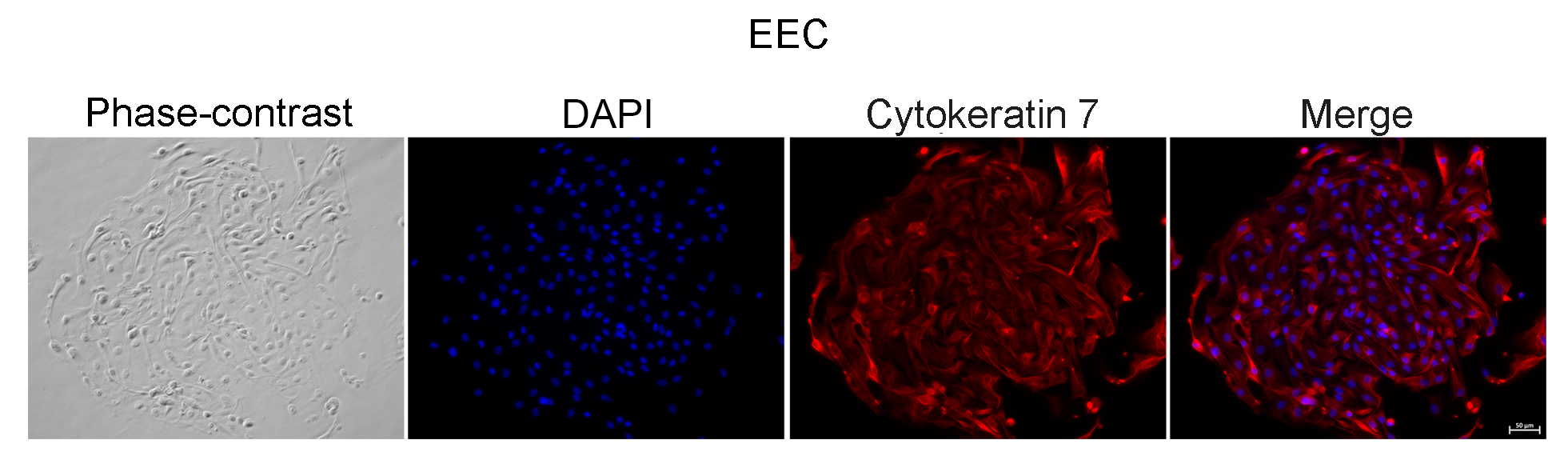

Supplement: FIGURE S5 — Morphology and Cytokeratin 7 staining of the cultured primary EECs. [file Image_5.TIF]

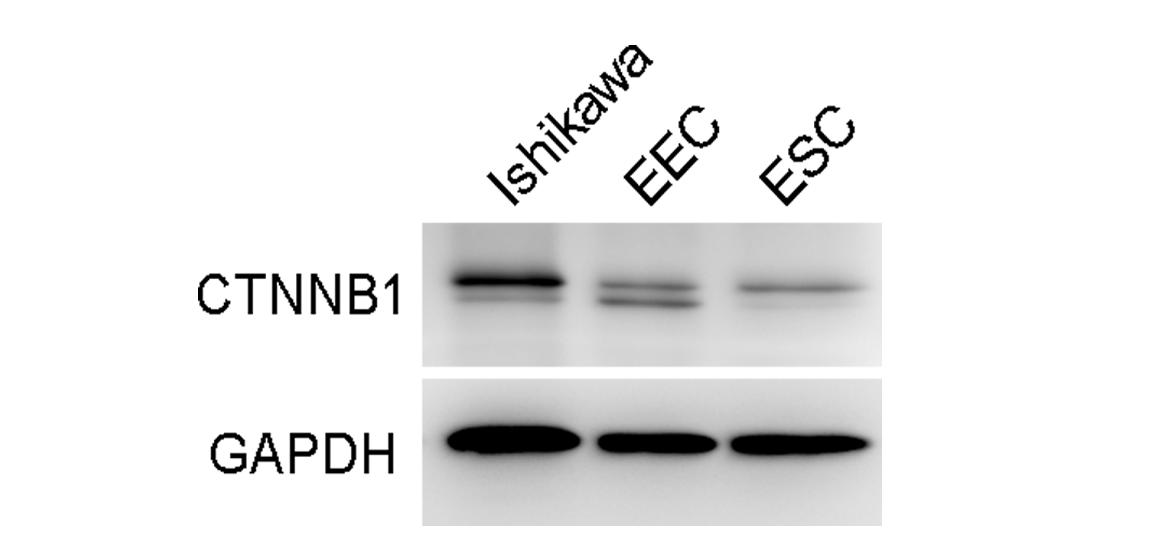

Supplement: FIGURE S6 — The differential expression of CTNNB1 isoforms in Ishikawa, EECs, and ESCs. [file Image_6.TIF]

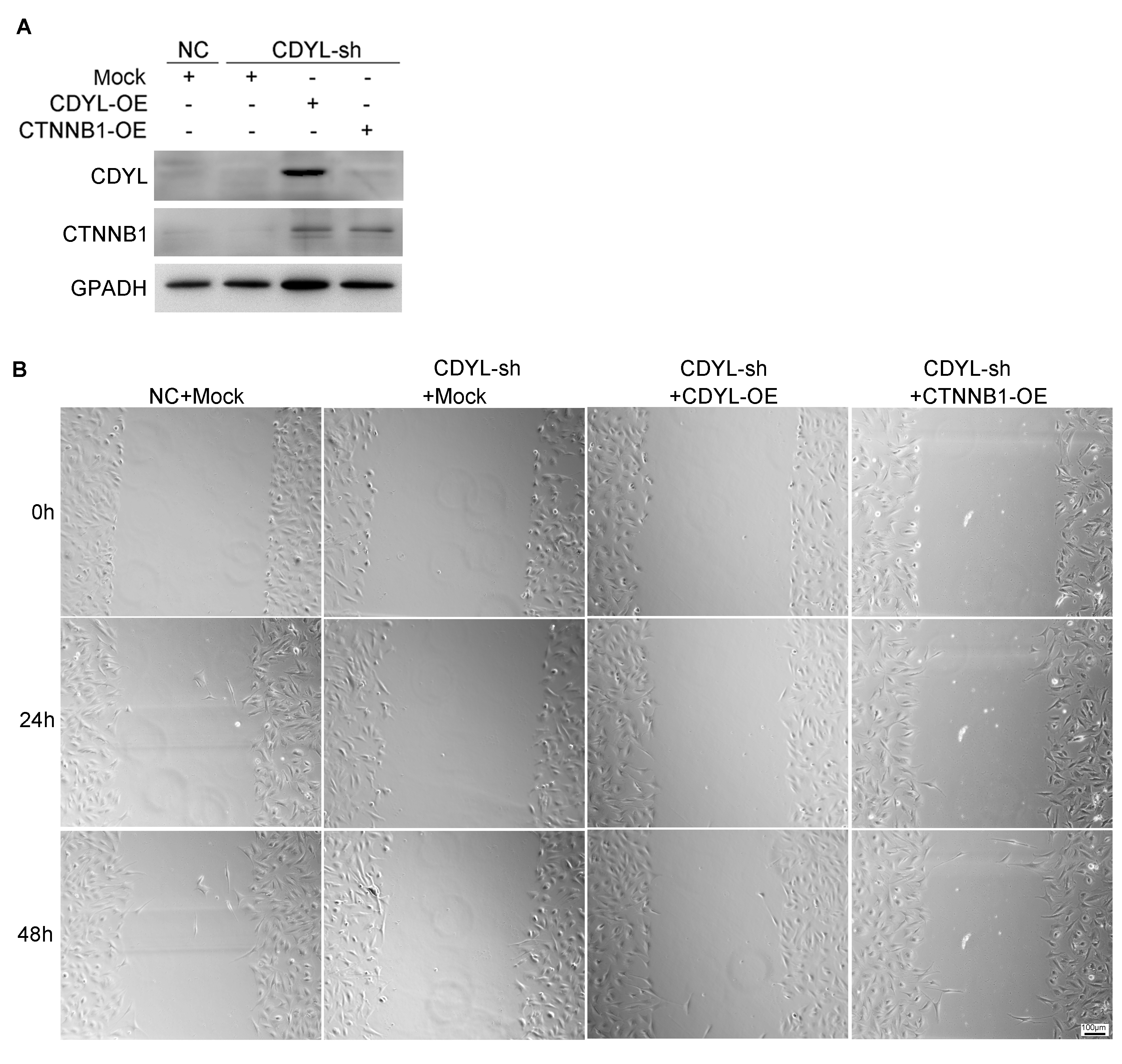

Supplement: FIGURE S7 — Effects of CDYL knockdown/over-expression on the expression of CTNNB1 and cell migration in primary endometrial stromal cells from RIF patients. (A) Effects of CDYL on CTNNB1 expression level in EECs by Western blot analysis. (B) Effects of CDYL on cell migration in ESCs by wound-healing assay. Bar = 100 μm. [file Image_7.TIF]

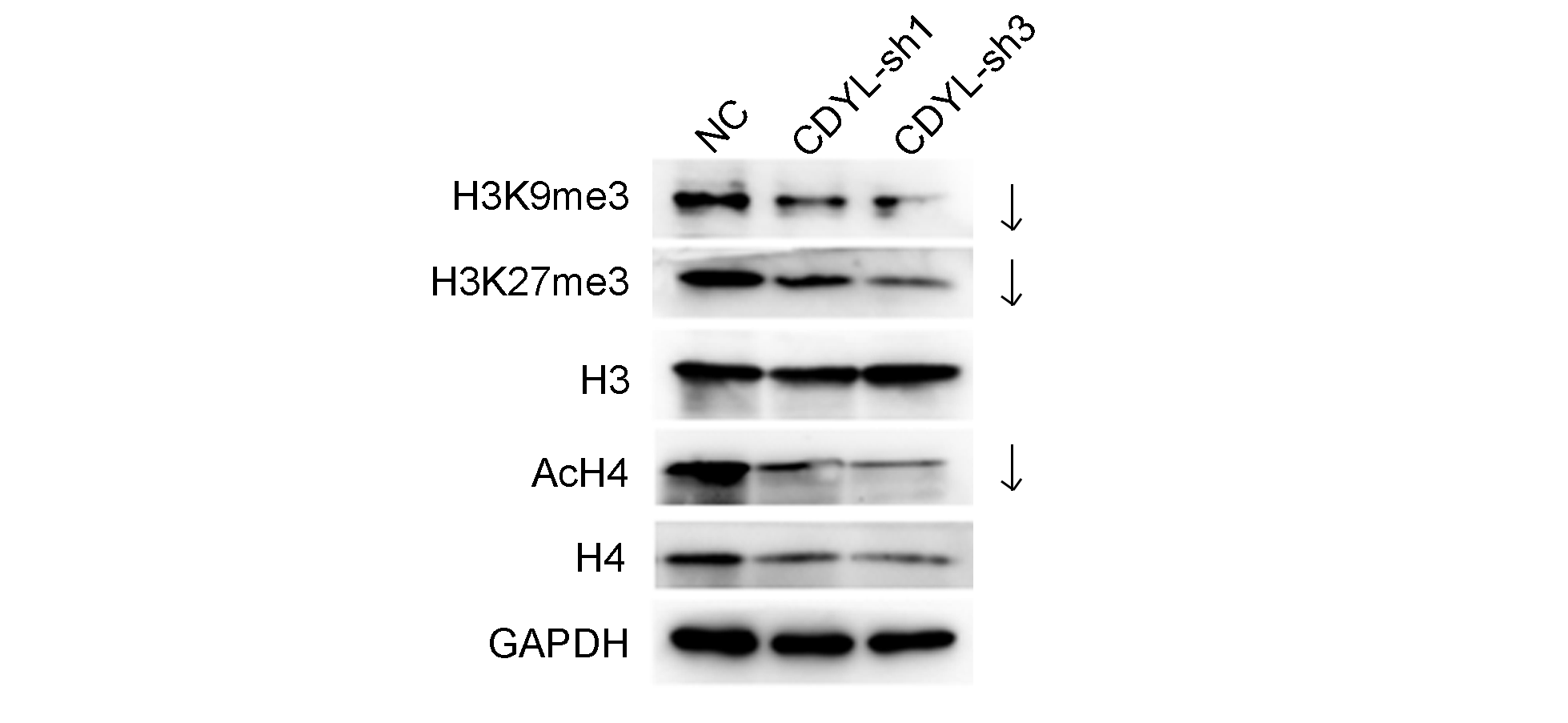

Supplement: FIGURE S8 — Detection of critical histone modifications in CDYL-sh Ishikawa cells. [file Image_8.TIF]

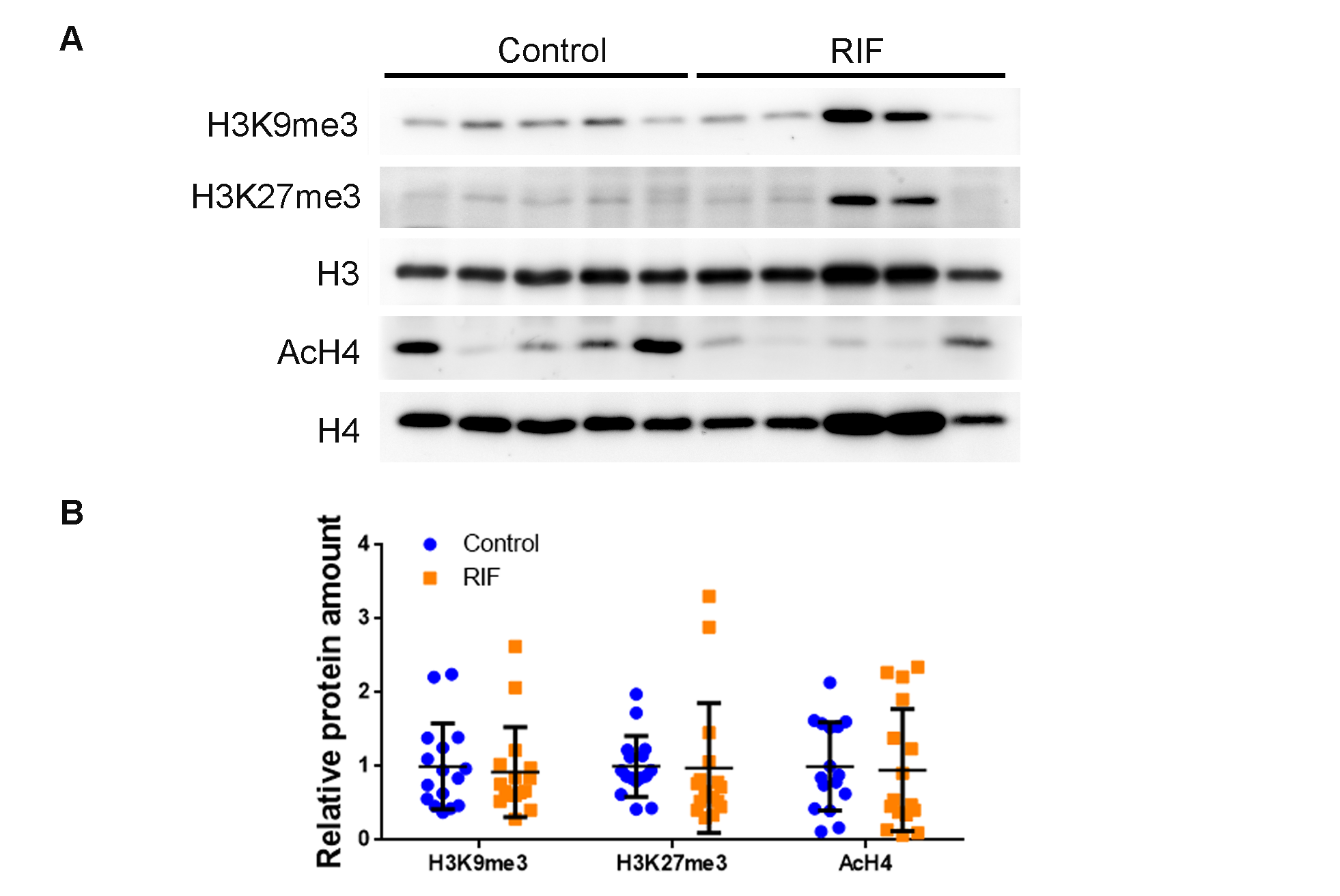

Supplement: FIGURE S9 — Detection of critical histone modifications in endometrial samples from control and RIF patients. (A) Representative results from western blot analysis. (B) Quantification of detected histone modifications in both control (n = 16) and RIF (n = 16) groups. [file Image_9.TIF]
