# Supplementary material for: Loss of CDYL Results in Suppression of CTNNB1 and Decreased Endometrial Receptivity
Source: Front Cell Dev Biol. 2020 Feb 25;8:105. doi: 10.3389/fcell.2020.00105 (PMC7051920; doi:10.3389/fcell.2020.00105)
Supplement: TABLE S1 — Primers used in this study. [file Table_1.docx]

**Supplemental data**

Table S1 *Primers used in this study.*

| Gene | Sequence (5’-3’) |
| --- | --- |
| CDYL-F | GCCGGTCGGAGCTTTATTG |
| CDYL-R | CGGAGATGTACCTTTCCCGTT |
| CTNNB1-F | CATCTACACAGTTTGATGCTGCT |
| CTNNB1-R | GCAGTTTTGTCAGTTCAGGGA |
| EPHA2-F  EPHA2-R | TGGCTCACACACCCGTATG  GTCGCCAGACATCACGTTG |
| GJA1-F | CAATCTCTCATGTGCGCTTCT |
| GJA1-R | GGCAACCTTGAGTTCTTCCTCT |
| IL6ST-F | CGGACAGCTTGAACAGAATGT |
| IL6ST-R | ACCATCCCACTCACACCTCA |
| JAG1-F  JAG1-R | GTCCATGCAGAACGTGAACG  GCGGGACTGATACTCCTTGA |
| MSX1-F | ACACAAGACGAACCGTAAGCC |
| MSX1-R | CACATGGGCCGTGTAGAGTC |
| NOTCH1-F  NOTCH1-R | GAGGCGTGGCAGACTATGC  CTTGTACTCCGTCAGCGTGA |
| PLA2G4A-F | ATGGATGAAACTCTAGGGACAGC |
| PLA2G4A-R | CTGGGCATGAGCAAACTTCAA |
| TGFB1-F | CAATTCCTGGCGATACCTCAG |
| TGFB1-R | GCACAACTCCGGTGACATCAA |
| SPHK1-F | GGCTGCTGTCACCCATGAA |
| SPHK1-R | TCACTCTCTAGGTCCACATCAG |
| VANGL2-F | ACCGCTCTAAGAGTCGAGATG |
| VANGL2-R | GTTACTACTGTCGTCGTTTCCC |
| GAPDH-F | TGACTTCAACAGCGACACCCA |
| GAPDH-R | CACCCTGTTGCTGTAGCCAAA |
