# Supplementary material for: Loss of CDYL Results in Suppression of CTNNB1 and Decreased Endometrial Receptivity
Source: Front Cell Dev Biol. 2020 Feb 25;8:105. doi: 10.3389/fcell.2020.00105 (PMC7051920; doi:10.3389/fcell.2020.00105)
Supplement: TABLE S2 — Antibodies used in this study. [file Table_2.docx]

**Supplemental data**

Table S2 *Antibodies used in this study.*

| Antibody | Dilution ratio | Product details |
| --- | --- | --- |
| Anti-CDYL rabbit polyclonal antibody | 1:600 | Novusbio, USA,  # NBP2-15851 |
| Anti-CTNNB1 mouse monoclonal antibody | 1:1000 | Abcam, USA,  # ab32572 |
| Anti-GAPDH rabbit monoclonal antibody | 1:1000 | Cell Signaling, USA,  #5174 |
| Anti-rabbit IgG, HRP-linked antibody | 1:3000 | Cell Signaling, USA,  #7074 |
| Anti-mouse IgG, HRP-linked Antibody | 1:3000 | Cell Signaling, USA,  #7076 |
