# Supplementary material for: Loss of CDYL Results in Suppression of CTNNB1 and Decreased Endometrial Receptivity
Source: Front Cell Dev Biol. 2020 Feb 25;8:105. doi: 10.3389/fcell.2020.00105 (PMC7051920; doi:10.3389/fcell.2020.00105)
Supplement: TABLE S3 — The effective target sequences for CDYL shRNA. [file Table_3.docx]

**Supplemental data**

Table S3 *The effective target sequences for CDYL shRNA.*

| ShRNA Name | Sequence (5’-3’) |
| --- | --- |
| Negative control | TTCTCCGAACGTGTCACGTAA |
| CDYL-sh1 | GCGTCTTCTGTTGTGGACTTGACTT |
| CDYL-sh2 | GCAAGATAATGGGAGGAGCATCTGCA |
| CDYL-sh3 | GAGATATTGTGGTCAGGAA |
